# Interleukin-17 Expression in the Barrett's Metaplasia-Dysplasia-Adenocarcinoma Sequence

**DOI:** 10.5402/2012/578149

**Published:** 2012-12-30

**Authors:** J. R. Bannister, A. L. Khan, D. W. Eccleston, R. K. Deol-Poonia, S. F. Hughes

**Affiliations:** ^1^Department of Biological Sciences, University of Chester, Parkgate Road, Chester, CH1 4BJ, UK; ^2^Aintree University Hospital, NHS Foundation Trust, Liverpool L97AL, UK

## Abstract

*Introduction*. This pilot study evaluated the expression of the proinflammatory cytokine IL-17 along the Barrett's metaplasia-dysplasia-adenocarcinoma sequence by establishing the expression levels of IL-17 in columnar epithelium, intestinal metaplastic cells, and dysplastic/glandular neoplastic cells. Immunohistochemical techniques were used to examine the accumulation of the proinflammatory cytokine IL-17 in forty (*n* = 40) formalin-fixed, paraffin-embedded oesophageal archived specimens across a range of endoscopic diagnostic categories, and a highly significant difference was found, where *P* ≤ 0.001, in IL-17 expression (Kruskall Wallis and Mann-Whitney *U*) between all the cell types examined. There was also a strong positive correlation (Spearman's rank correlation) between disease progression and IL-17 expression (*r*_*s*_ = 0.883, *P* < 0.001, *n* = 29), IL-17 expression was absent or absent/weak in columnar epithelium, weak to moderate in columnar metaplastic cells, and moderate to strong in dysplastic/neoplastic cells, which demonstrated that the elevation of IL-17 expression occurs in the progression of the disease. Understanding the differential expression of IL-17 between benign and malignant tissue potentially has a significant diagnostic, prognostic, and therapeutic value. Ultimately, this selective biomarker may be employed in routine clinical practice for the screening of oesophageal adenocarcinoma.

## 1. Introduction

Oesophageal Adenocarcinoma (OAC) is the focus of intense research into the cause of the disease and treatment [1]. In the context of OAC screening, a biomarker capable of reliably predicting progression to dysplasia or cancer by increased expression is highly valuable [2]. Oesophageal cancer develops symptoms late in the course of this disease and carries a poor prognosis. In the UK, oesophageal cancer is the eleventh most commonly diagnosed cancer and, of greater concern, the sixth most common cause of death from cancer, which represented 1 : 20 of all cancer deaths in the UK; 8,161 new cases were diagnosed in 2009 and the mortality rate was 7,610 in 2010 [3].

The role of inflammation in the development of OAC is not well understood, unlike its role in colon and breast cancers. However, this role in the progression of OAC is now beginning to gain greater attention [4]. Proinflammatory cytokines and their pathways are associated with inflammation in Barrett's oesophagus and tumourigenesis. Elucidating the association between inflammation and OAC may contribute to the eventual prevention of OAC [5]. Further assessment of these cytokines has recently been recommended, as these may be important targets for early diagnosis and chemoprevention drugs [6, 7].

Inflammatory factors are commonly associated with contributing to the development of tumours; chronic inflammation is often associated with malignancy [8]. Abdel-Latif et al. [5] concluded that chronic inflammation plays a crucial role in the pathogenesis of the disease where inflammatory cytokines are linked with advancement of tumourigenesis. IL-17 induces a wide variety of factors, which include chemokines, cytokines, acute phase proteins, tissue remodelling proteins, antimicrobial proteins, and nitric oxide. Cytokines that induce IL-17 include IL-6 and IL-8, and expression increases in both as the disease progresses. *β*-defensin, indirectly induced by IL-17, recruits dendritic cells and T lymphocytes to the inflamed site in the oesophagus through interaction with chemokine receptor (CCR) 6. Chemokine (C-C motif) ligand (CCL) 20 is up-regulated by IL-17, a chemokine that also attracts dendritic cells and T lymphocytes to the inflamed site. *β*-defensins and CCL20 serve to recruit and extend the T_H_17 population at the inflamed site [9]. IL-17 is implicated in several chronic inflammatory diseases, including the autoimmune diseases, rheumatoid arthritis (RA), and psoriasis [10, 11]. 

Recent evidence suggests that the presence of infiltrating IL-17+ cells, which include lymphocytes, mast cells, and neutrophils, in patients with OAC, may have a pathogenic role in this disease. Several studies have demonstrated a high frequency of IL-17+ cells in inflammation-related cancers including OAC [12], nonsmall cell lung carcinoma, where IL-17 has been implicated in metastasis of lung cancer by promoting lymphangiogenesis [13] and hepatocellular carcinoma where IL-17 is also believed to promote angiogenesis [14]. 

The aim of this pilot study was to evaluate the expression of the proinflammatory cytokine IL-17 along the Barrett's metaplasia-dysplasia-carcinoma sequence by establishing the expression levels of IL-17 in columnar epithelium, intestinal metaplastic cells, and dysplastic/glandular neoplastic cells.

## 2. Materials and Methods

**Ethics Statement**

This study, which included the use of archived human tissue samples, was approved in writing by the Proportionate Review Sub-Committee of the National Research Ethics Service Committee East Midlands-Nottingham 2 Research Ethics Committee in the United Kingdom, reference 11/EM/0384, on November 23 2011. Written permission for this study was also received from the Research and Development Directorate at Aintree University Hospital National Health Service Foundation Trust, reference 451/11, on November 28 2011.

Forty distal oesophageal biopsy samples embedded in formalin-fixed paraffin blocks, originally collected between 2010 and 2011, were retrieved from the Cellular Pathology department archive at Aintree University Hospitals National Health Service Foundation Trust, UK; ten from each of the following endoscopic examination result categories: Normal oesophagus, reflux oesophagitis, Barrett's oesophagus, and oesophageal adenocarcinoma. The mean age of the patients involved with this study was 71 years (range: 49–88).

The IL-17 expression in columnar epithelium, columnar metaplastic cells, and dysplastic/glandular neoplastic cells was compared. A negative control was established using an internal control on each slide (squamous mucosa), and a positive control was established, which was taken from normal kidney tissue. Immunohistochemistry was with IL-17 antibody, and counter stained with haematoxylin.

The rabbit polyclonal antibodies against human origin IL-17 sc7927 from Santa Cruz Biotechnology (SCBT) have been used extensively in recent studies, including human immunohistochemical studies; therefore, this IL-17 antibody was selected for use by this study. The dilutions 1 : 50, 1 : 100, and 1 : 200 of the IL-17 antibody were prepared as recommended by SCBT, and the stained tissue sections were examined via microscopy to find the initial optimum dilution, which was 1 : 50. Further dilutions were prepared, which included 1 : 30, 1 : 40, 1 : 50, and 1 : 60, and of these, the 1 : 40 dilution was the optimum to visualise IL-17 expression in the patient tissue samples. The same batch of this primary antibody was used throughout. Sample sections were cut at 2 *μ*m. All positive controls were taken from a human kidney biopsy [15].

Prior to staining, the antigens were unmasked at 97°C with a target retrieval solution at pH 9.4. 30 mL of Dako EnVision FLEX Target Retrieval Solution (High pH 50x) was added to 1.5 L of distilled water and treated at 97°C for 20 minutes using the Dako PT link machine (Dako).

The primary IL-17 antibody (1 : 40 dilution in Dako REAL Antibody Diluent, Santa Cruz Biotechnology, USA) was used in this study. Staining was conducted using Dako Autostainer Link 48 (Dako), which added 300 *μ*L of undiluted Dako EnVision FLEX Peroxidase-Blocking Reagent to each slide for 5 minutes. 300 *μ*L of diluted IL-17 antibody was subsequently added to each slide for 30 minutes. The treatment continued by adding 300 *μ*L of undiluted Dako EnVision FLEX+ Rabbit (Linker) to each slide for a further 15 minutes. After the Linker treatment, 300 *μ*L of undiluted Dako EnVision FLEX/horseradish peroxidase (HRP) was added to the slides for 20 minutes. This was followed by adding a 300 *μ*L of diluted chromogen reagent to each slide for 5 minutes, followed by a buffer wash, and then by a second application of 300 *μ*L of diluted chromogen for 5 minutes. The diluted chromogen was prepared by placing 20 drops of Dako EnVision FLEX DAB+ Chromogen into 20 mL of Dako EnVision FLEX Substrate Buffer. The final step of the automated autostainer process was to add 300 *μ*L of Dako EnVision FLEX Haematoxylin (Link) to each slide for 5 minutes. Between each treatment, the slides were washed with a diluted wash buffer. The diluted wash buffer consisted of 0.5 L Dako EnVision FLEX Wash Buffer (20x) and 10 L of distilled water. This buffer was placed into a large container that was connected to the Autostainer.

Before the cover slips were mounted, the sections were dehydrated by the Leica ST4040 system (Leica). The sections were dehydrated in ascending alcohols (50%, 70%, and 99% twice) for 15 seconds each using Alcohol Methylated Spirit 99% (Genta Medical) diluted with distilled water. The system treated the slides with Xylene (Genta Medical) for 5 minutes. After the section dehydration, the slides were placed into the automated cover slipper, the Leica CV5000 (Leica).

## 3. Histological Evaluation of IL-17 Staining

Two experienced pathology laboratory staff independently assessed tissue sections for the staining intensity of IL-17, with scoring from 0 to 3 (0—no staining, 1—week intensity, 2—medium intensity, and 3—high intensity). 

The One-way nonparametric ANOVA (Kruskal-Wallis test), and the Mann-Whitney *U* test were used to determine if there was a difference in IL-17 expression. Pairwise comparisons were made between the columnar epithelium, intestinal metaplastic cells, and dysplastic cells/glandular neoplastic cells. The association between disease progression and the level of expression of IL-17 was evaluated using Spearman's rank correlation method to determine whether there was an association between disease progression and the expression of IL-17. The original Haematoxylin and Eosin (H&E) slides were used to compare the morphological features of the samples with the IL-17 staining.

## 4. Results

**Figure 1:**
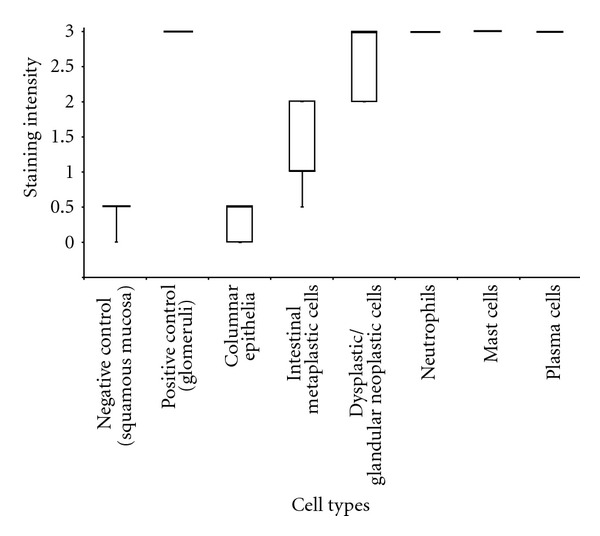
Boxplot of the IL-17 staining intensity scores. Demonstration of the median (bold horizontal line), interquartile range (box where given) and whiskers to highest and lowest levels. Negative controls (*n* = 16), positive control (*n* = 8)—note: 1 positive control per batch run, columnar epithelium (*n* = 7), intestinal metaplastic cells (*n* = 11), dysplastic/glandular neoplastic cells (*n* = 11), neutrophils (*n* = 6), mast cells (*n* = 7), plasma cells (*n* = 1).

**Figure 2:**
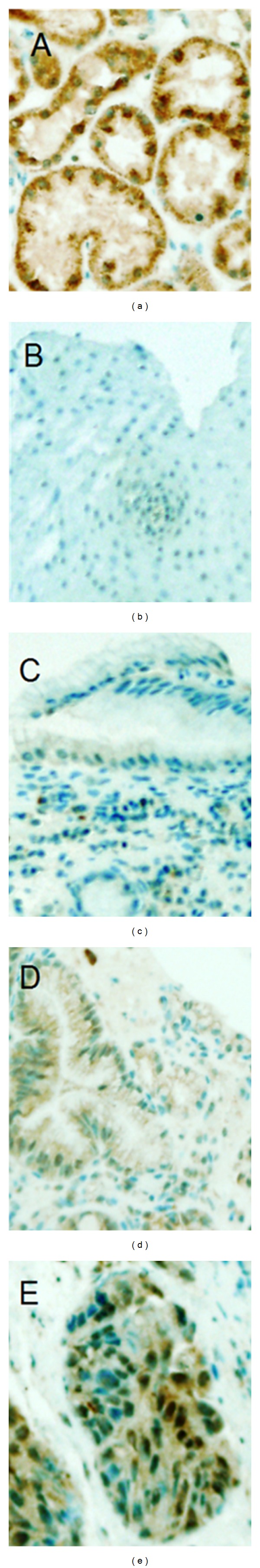
Expression of interleukin-17. (a) is an IL-17 positive control (human kidney). The epithelium lining the kidney collecting ducts was strongly stained. Magnification ×100. (b) is an IL-17 negative control and demonstrates IL-17 immunohistochemistry treatment within the oesophageal squamous mucosa. IL-17 staining is brown when present. Magnification ×200. (c) demonstrates IL-17 expression in the columnar epithelium (without intestinal metaplasia) where IL-17 staining was absent/weak. Magnification ×200. (d) demonstrates IL-17 expression in the intestinal metaplasia where the IL-17 stain intensity was moderate. Intestinal metaplastic cells surround the goblet cells. Magnification ×100. (e) demonstrates IL-17 expression in the glandular neoplastic cells where IL-17 stain intensity was strong. IL-17 staining was absent within the neighbouring squamous mucosa region. Magnification ×200.

Expression levels of IL-17 in columnar epithelium, intestinal metaplasia, and dysplasia/glandular neoplasia were compared. There was significant difference between the expression of IL-17 between the three different cell types (columnar epithelium, intestinal metaplastic cells, and dysplastic/glandular neoplastic cells), (*X* ^2^ = 21.852, *df* = 2, *P* < 0.001, *n* = 29, Figure 1). Squamous epithelium included in the paraffin-embedded blocks demonstrated absent cytoplasmic staining and acted as an internal negative control (Figure 2). Kidney-tissue positive controls demonstrated strong IL-17 expression with the collecting ducts (Figure 2). At the optimal concentration, columnar epithelium demonstrated absent IL-17 staining in 3 of 7 (43%) or absent/weak staining (Figure 2) in 4 of 7 (57%). Interestingly, the staining was distributed throughout the cytoplasm, and the stains were uniform for all the cell types examined (*n* = 29). For the 11 intestinal metaplasia samples, 5 of 11 (46%) demonstrated moderate staining (Figure 2), 4 of 11 (36%) demonstrated weak staining, and 2 of 11 (18%) demonstrated absent/weak staining. These staining scores were elevated when compared with columnar epithelium, as demonstrated by the staining intensity scores, and the difference was highly significant difference (MWU, *Z* = −3.242, *P* = 0.001, *n* = 18). For the 11 dysplasia and glandular neoplasia regions, 6 of 11 (55%) demonstrated strong staining (Figure 2), 4 of 11 (36%) demonstrated moderate staining, and 1 of 11 (9%) demonstrated moderate/strong staining. These staining scores were elevated when compared with columnar epithelium, and also with intestinal metaplasia; these were (MWU, *Z* = −3.598, *P* < 0.001, *n* = 18) and (MWU, *Z* = −3.483, *P* < 0.001, *n* = 22), respectively. Neutrophils (*n* = 6) and mast cells (*n* = 7) found in normal, Barrett's oesophagus, and OAC cases were all strongly positive. Surprisingly, only one case of plasma cells (*n* = 1) was found in an oesophagitis case, and it was strongly positive.

The Spearman rank correlation coefficient (non-parametric correlation analysis method) revealed that there was a highly significant correlation (*r*_*s*_ = 0.883, *P* < 0.001, *n* = 29) between IL-17 expression and the progression of the disease, where the disease progresses stepwise from columnar epithelium to intestinal metaplasia, and onwards towards dysplasia and glandular neoplasia. There was a strong positive correlation between disease progression and IL-17 expression, and this suggests that IL-17 expression increases as the disease progresses.

Immunohistochemistry staining scores were validated by comparing the scores between two independent observations. The Cohen's Kappa test results indicated that there was “almost perfect agreement” (*k* = 1.00, *n* = 29) between the intensity scores provided by Dr. Abdul Khan and Mr. David Eccleston, both experienced histology pathology staff at the Aintree University Trust Hospital. Their long working relationship together within the Trust may have led to the high scoring concordance.

## 5. Discussion and Conclusion

The findings, that there is a highly significant increase in IL-17 expression between columnar epithelium and intestinal metaplastic cells, and between intestinal metaplastic cells and dysplastic/glandular neoplastic cells in the distal oesophagus, are novel (Figure 1). Amongst the native squamous oesophagus and oesophageal columnar epithelium, there was little or no expression of IL-17. The trigger for IL-17 elevated expression is not clear. There appears to be a stage in the transition between columnar epithelium to intestinal columnar epithelium where expression of IL-17 becomes apparent. During the investigation some IL-17 staining of the nuclei, but not cytoplasm, was observed in the columnar epithelium and squamous mucosa; this may be an indication of the transition phase where IL-17 becomes apparent.

TH_17_ cells secrete IL-17. TH_17_ cells are associated with mucosal immunity, particularly within the gastrointestinal tract, and autoimmune diseases. A study examining gastric cancer conducted by Zhang et al. [15] found IL-17 mRNA expression in the majority of the tumour samples, and expression was not detected in the patient's normal gastric tissue. This concurs with the finding of this study in that IL-17 expression is elevated in tissue affected by OAC at a level significantly greater level than that found in normal tissues. Radosavljevic et al. [16] studied serum IL-17 levels in patients with colorectal carcinoma and found significantly higher IL-17 serum levels in patients with colorectal carcinoma than found in healthy subjects. Their study indicated that IL-17, combined with other serum markers, is a valuable tumour marker in patients with colorectal carcinoma, which may provide additional information about the characteristics of the tumour. Examining similar serum markers against a possible case of Barrett's oesophagus or oesophageal adenocarcinoma may also provide valuable diagnostic and prognostic information, which offers the prospect of an alternative cost-effective and noninvasive approach to the clinician.

Recent research suggests that there is a diverse range of IL-17 producing cells amongst the immune system community, and innate immunity cells may secrete IL-17 [17]. Mast cells and neutrophils were found in normal, Barrett's oesophagus, and OAC cases. Mast cells and neutrophils were associated with strong IL-17 expression in this study, suggesting that these innate immune cells have an active role in maintaining high levels of IL-17 amongst the disease-affected tissues. Lin et al. [18] studied the pathogenesis of psoriasis and demonstrated that mast cells and neutrophils, rather than T lymphocytes, are the prominent immune cell types that express IL-17 in the human skin. Release of IL-17 from mast cells and neutrophils may be important to the pathogenesis of OAC and may have a similar pattern of chronic inflammation as seen in psoriasis.

Clear evidence of any lymphocyte involvement in the normal, Barrett's oesophagus, or OAC cases was not found. However, the stroma was affected by strong background staining, and it was difficult to differentiate between stroma and inflammatory cells via microscopy. Difficulties were also experienced in attempting to make this distinction in the corresponding archived haematoxylin and eosin stained slides; there was significant background staining in most of these cases, and because of these problems, the stroma regions found in the cases examined were not included in the results. Reducing the concentration of the IL-17 antibody may have aided interpretation within the stroma, but at the expense of being able to interpret regions outside the stroma; these regions (outside the stroma) would deviate from their optimum concentration.

This study demonstrated that IL-17 expression increases as the disease progresses along the metaplasia-dysplasia-carcinoma sequence, which potentially has a significant diagnostic, prognostic, and therapeutic value. Understanding the differential expression of IL-17 between benign and malignant tissue may allow diagnostic, prognostic, and therapeutic strategies to be developed. The evidence presented by this study suggests that IL-17 has a role in the development of OAC, which merits further investigation. 

## Abbreviations

IL: Interleukin
MWU: Mann-Whitney *U* test
OAC: Oesophageal adenocarcinoma
RA: Rheumatoid arthritis
UK: United Kingdom.
